# Insufflation of Medicated Powders in Gleet

**Published:** 1866-10

**Authors:** 


Insufflation of Medicated Powders in Gleet.—Dr. Mallez has contrived an instrument by which medicated powders can be conveyed to the membraneous portion of the urethra, a region where lies the cause of the gleet, and which region ordinary injections cannot reach. The instrument is chiefly composed of a catheter opened at both ends, another which glides into it, and an apparatus for receiving and projecting the powders. M. Ricord presented the instrument to the Academy of Medicine of Paris, and cases are mentioned in which excellent results were obtained by insufflating subnitrate of bismuth. It is plain that, by previous micturition, the powder may remain hours in the passage; and it is thought that the vagina, uterus, or any fistulous tract may perhaps be reached in the same way.—*Lancet.*

